# A New Philosophy for the Margin of Appreciation and European Consensus

**DOI:** 10.1093/ojls/gqab031

**Published:** 2021-09-29

**Authors:** Bosko Tripkovic

**Keywords:** European consensus, European Court of Human Rights, European Convention on Human Rights, human rights, margin of appreciation

## Abstract

The article advances an anti-foundationalist account of the key doctrines of the European Court of Human Rights (ECtHR): the margin of appreciation (MoA) and European consensus (EuC). The first part of the article argues that anti-foundationalism, which understands the existence of human rights as ultimately dependent on social practices and their justification as based on a plurality of values, is a credible conception of human rights grounds. The second part contends that anti-foundationalism offers the best explanation of the MoA and EuC, without making the ECtHR’s practice less normatively appealing. These arguments challenge the dominant critiques of the MoA and EuC, which often assume, but rarely explicitly defend, a foundationalist understanding of human rights. While the ECtHR’s use of the MoA and EuC can be inadequate, this is not because it is mistaken about the grounds of human rights.

## 1. Introduction

It matters how the European Court of Human Rights (ECtHR) understands the nature of human rights. The question is of obvious practical importance: each year, the ECtHR decides thousands of cases based on its conception of human rights. But the issue is also of immense theoretical significance: philosophical accounts of human rights cannot disregard the fact that the ECtHR has developed one of the most elaborate practices of human rights protection in the world. The practical and theoretical aspects of this question are related, for it would not only be theoretically extraordinary if it turned out that the Court were profoundly mistaken about the nature of human rights, but it would also follow that the practical weight of its judgments, based on a flawed conception of human rights, should be reconsidered. Yet, this conclusion sometimes seems unavoidable.

The reason for this are two foundationalist assumptions about the grounds of human rights. The first is that the existence of human rights is not ultimately contingent on social practices, and the second is that their justification exhaustively depends on valuable features of human beings.^1^ On the foundationalist view, the role of the ECtHR is to discover practice-independent requirements of human rights without ascribing significant normative weight to considerations external to the protected features of human beings.^2^ As a consequence, foundationalists face difficulties in explaining the key legal doctrines of the ECtHR, such as the margin of appreciation (MoA) and European consensus (EuC), which determine the normative content of human rights based on contingent social practices, and reasons which do not exclusively pertain to valuable features of human beings. Given the centrality of these doctrines, this leads them to a counter-intuitive conclusion that one of the leading human rights institutions in the world is fundamentally and persistently mistaken about the nature of human rights.

In this article, I argue for an anti-foundationalist account of the grounds of human rights and show that it can explain the MoA and EuC without making the ECtHR’s practice less normatively attractive. In the first part, I contend that human rights ultimately exist in virtue of contingent human rights practices,^3^ and that their justification does not exclusively depend on valuable features of human beings.^4^ In the second part, I argue that anti-foundationalism better accounts for the MoA and EuC, without undermining the protection of minorities or the universality of human rights. Taken together, these arguments show that the foundationalist critique of the MoA and EuC is not well-founded.

## 2. The Grounds of Human Rights

The grounds of human rights are the more fundamental features of reality in virtue of which human rights exist.^5^ For example, the right to life may be thought to exist in virtue of the value of human dignity,^6^ freedom of expression because of the value of human agency or autonomy,^7^ and rights to a fair trial and political participation, among other things, in virtue of certain institutional properties of contemporary societies.^8^ In each of the examples, the existence of a human right is explained with reference to a more basic entity upon which it depends, and without which there would be no such right.^9^ Such dependence pertains to fundamentality and not causality:^10^ for instance, human dignity is not a previous event or action which has brought about the right to life as its effect, but may, as a more fundamental entity, generate such a right, as a less fundamental entity.^11^ Foundationalism and anti-foundationalism offer competing conceptions of human rights grounds.

The key claim of foundationalism is that human rights are grounded in our humanity,^12^ and that they belong to human beings ‘as such’.^13^ This claim is then cashed out in terms of moral human rights,^14^ which are said to exist in virtue of valuable features of human beings, such as their agency, interests or needs.^15^ The grounding relation between valuable features of human beings and moral human rights is crucial, for it underpins the foundationalist understanding of universality of human rights. According to foundationalism, human rights are both diachronically and synchronically universal: they have existed ‘at all times and in all places’, even in a ‘state of nature’,^16^ and independently of ‘any actual institutional or social recognition’.^17^ The idea is that because human rights are grounded in valuable features of human beings—which purportedly do not vary across space and time—such rights belong to everyone, and are, in that sense, universal.^18^

This conception of universality motivates two foundationalist theses about the grounds of human rights. The first relates to their mode of existence. For foundationalism, the existence of human rights is at the most fundamental level independent from contingent social practice(s).^19^ If human rights are diachronically and synchronically universal, they must inherit such universality from their grounds, and the most fundamental grounds of human rights then also need to be independent from contingent and evolving frameworks of evaluation embedded in social practices.^20^ Note that this is a thesis about the ultimate grounds of human rights. Foundationalism does not exclude the possibility that contingent facts or entities can play a role in the grounding base of human rights if they acquire such a role in virtue of more basic moral human rights, or valuable features of human beings which underlie such moral human rights.^21^ For example, some human rights, such as the right to a fair trial or the right to education, may partly depend on contemporary institutional practices, as long as they are ultimately derived from more fundamental and practice-independent moral grounds.^22^

The second thesis relates to the justification of rights. Foundationalism confines the most fundamental grounds of human rights to normative considerations which pertain to valuable features of human beings.^23^ This view of justification of human rights is not entailed by their purported practice-independent existence. It is possible to understand the existence of rights as practice-independent, and accept a wider range of values as human rights grounds.^24^ But foundationalist views attach the ultimate grounds of human rights to the valuable features of human beings only:^25^ as explained, because these features are seen as immutable and independent from contingent social practices, this allows foundationalism to account for diachronic and synchronic universality of rights and the status of all human beings as right-holders.^26^

Anti-foundationalism denies the two foundationalist theses.^27^ The first anti-foundationalist thesis is that the existence of human rights is not ultimately practice-independent. According to anti-foundationalism, the existence of human rights, at the most fundamental level, depends on social practices, which, in turn, ground normative values that justify human rights.^28^ Human rights exist in virtue of some normative framework embedded in contingent social practices which holds that there is a normative justification for human rights. Note that anti-foundationalism does not deny the universality of human rights: it is enough that evaluative concern towards all human beings—which is important enough to justify the duty to protect their interest, status or needs in the form of human rights—obtains from that evaluative framework.^29^ The universality of human rights is thus seen as a normative commitment, while the existence of such commitment is owed to contemporary social practices.^30^

The second anti-foundationalist thesis is that the justification of human rights need not be based on valuable features of human beings only. This thesis does not follow directly from the anti-foundationalist understanding of the existence of rights. But—as I shall explain—because anti-foundationalism is not wedded to the view of universality which attaches human rights to features of human beings, its account of justification of human rights can include a wider range of normative values:^31^ what matters is that justification of rights both reflects and strikes the appropriate balance between normative considerations internal to human rights practice.^32^ In what follows, I will argue that anti-foundationalism better explains the existence of human rights, and then I will draw implications of this explanation for their justification.

### A. Existence

To compare these approaches, it is useful to focus on the question of diachronic existence of human rights.^33^ A grounding framework should be able to explain two intuitions. First, some claims about the universal diachronic existence of human rights do not appear to be true: for instance, the proposition that the right to a fair trial or free elementary education of cave-dwellers existed seems false. This is so for a number of reasons, including the fact that cave-dwellers did not think of themselves as having such rights, there was no political authority to claim such rights against, there were no institutions to which the content of such rights would pertain and—even if cave-dwellers had a rudimentary notion of a right to fairness or minimal educational provision—it is doubtful that these would apply beyond the immediate social group in a way in which human rights normally apply.^34^ Secondly, some claims about human rights do seem to export across time, as it is meaningful to say that, for example, Genghis Khan’s invasions violated human rights of the civilian population.^35^ This claim is intelligible in spite of the lack of human rights vocabulary at the time, and seems to hold even for rights which could be seen as less central.^36^ Anti-foundationalism has certain theoretical and normative advantages in explaining these intuitions.

Foundationalism can respond to the first category of claims in two ways. First, it can accept that some human rights of cave-dwellers did not exist, but argue that a more basic moral human right or principle—which can ground less fundamental rights, such as the right to a fair trial or free elections—existed at the time.^37^ On this view, contemporary institutional practices simply implicate the more basic right or principle and thus generate the less basic right. But this explanation does not sit well with the foundationalist conception of human rights grounds. Take the example of the right to a fair trial. Even if cave-dwellers had a basic conception of a right to be treated fairly, there would still be an across-the-board failure throughout much of history to notice its central deontic implications, one of the main ones being the duty to establish independent and impartial institutions that would secure the right to a fair trial, such as courts.^38^ The problem is that this explanation reverses the grounding relation at the core of foundationalism: it is not the case that the right to a fair trial is simply triggered by the existence of contemporary institutions, which provide a new context in which the right to fairness applies; instead, the requirement to establish such institutions should follow from the right to be treated fairly, based on a human interest in fair treatment which presumably obtains at all times. The same holds for many other rights, such as the right to free elections or education, as they all incorporate positive duties to create institutions that make their protection and enjoyment possible.^39^ And it does not help to argue that the valuable features of human beings have changed over time to explain the lack of recognition of such rights: on the one hand, interests in fair treatment, political equality or knowledge presumably do exist diachronically; on the other, if valuable features of human beings do change over time and give rise to new rights, this undermines the foundationalist claim that human rights exist diachronically in virtue of such features.^40^

Secondly, foundationalism can accept the implication that human rights existed but were not recognised by humans: if the existence of human rights is ultimately practice-independent, it is possible for all participants in human rights practices to be mistaken about human rights. Indeed, humans have not, until very recently, recognised the existence of human rights or been able to work out even their most central implications. For example, they have been mistaken—by our standards—about a range of key human rights requirements, pertaining to, for example, gender equality or LGBTQ+ rights, or their paradigmatic structural features, such as their cosmopolitan character.^41^ But the question then becomes how we can reliably track this realm of practice-independent human rights. The difficulty is to explain why we are able to access the domain of practice-independent human rights while such access was not available to our ancestors.^42^

In offering such an explanation, foundationalism needs to demonstrate that our current epistemic access to the domain of practice-independent human rights is more reliable.^43^ It is not clear what such an account would look like: as noted by others, the foundationalist notion of derivation of rights from their grounds must be abstract enough to be grossly misapplied, while at the same time it needs to be specific enough to generate an attractive list of particular human rights.^44^ But even if this problem can be overcome, it is difficult to see how such an account would be verified. On the one hand, if the account relies on beliefs about human rights that obtain in our current human rights practices, such beliefs can also be in radical error in relation to the realm of practice-independent rights and thus equally unreliable.^45^ The cave-dwellers could then be, from the perspective of their own practice-dependent beliefs, similarly justified in thinking that a range of important human rights did not actually exist. On the other hand, it is not open to foundationalism to argue that such epistemic access is based on the grounds of human rights which are self-evident, or that practice-independent verification is not necessary. The former approach would treat beliefs about human rights which are subject to controversy, such as the belief that human rights are grounded in valuable features of human beings, as axiomatic.^46^ The latter would allow the normatively hazardous gap between the realm of human rights practices and practice-independent human rights to persist, leaving open the possibility that our practices are in radical error in relation to human rights requirements, and that we can be completely misguided about the true normative requirements of human rights.^47^

There could be other ways out of this conundrum,^48^ but the problems facing foundationalism seem significant enough to warrant an analysis of the possible anti-foundationalist solution.^49^ Recall that the anti-foundationalist account of human rights grounds makes them ultimately practice-dependent, which suggests that human rights of cave-dwellers did not exist. This view may, however, lead to obstacles in explaining the second intuition identified above, which implies that normative judgments about human rights do export across time; moreover, should this indeed be the case, anti-foundationalism could undermine our ability to normatively assess contexts in which human rights are not accepted in contingent social practices. Let us see if anti-foundationalism can overcome these obstacles.

The first obstacle is less problematic. Because anti-foundationalism grounds the existence of human rights in social practices, for these exporting intuitions to be meaningful it only needs to be the case that our contemporary practices do in fact ascribe rights to human beings in a way which holds across times and places.^50^ And the way in which we speak of human rights in practice does not presuppose that they apply to others only if they also accept the basic precepts of human rights, but that human rights apply notwithstanding their attitudes. For example, the prohibition of torture applies not only to cultures which accept it, but to all cultures, irrespectively of their recognition of the impermissibility of torture. In other words, social practices which ground human rights determine the perspective of evaluation and not of the evaluated.^51^ In the same way, our practices ascribe value to human beings irrespectively of the historic period they lived in, and this is what enables us to meaningfully talk about there being human rights violations even if humans were not aware of such rights at the time.^52^

The key concern, however, is whether this explanation undermines the normative authority of human rights. I want to suggest that anti-foundationalism need not undercut the critical potential of human rights in problematic ways and that, in fact, there may be some normative advantages to explaining human rights in anti-foundationalist terms.^53^ Normative practices are complex, sensitive to their purposes, and allow for coherence-based reasoning: it is possible to conclude that a part of human rights practice does not advance the purpose of the practice as a whole, or that it is inconsistent with a more general principle accepted in the practice.^54^ And because human rights occur, as Beitz puts it, at the ‘middle level of practical reasoning’,^55^ and because they do not consume our moral universe completely, they are answerable to a range of more fundamental moral concerns. At the same time, the recognition of the contingency of human rights need not lead to practical inaction. The fact that human rights are practice-dependent need not imply illegitimacy of interference when the central values of such practice are violated.^56^ Rather, it demands a careful reflective exercise to determine the boundaries of the core evaluative commitments, which includes both the notion of respect for differences and protection of values at the centre of a common and evolving evaluative framework.^57^ And this careful approach to imposing our understanding of human rights on others should not be seen as a limitation of anti-foundationalism, but as its advantage.^58^

Moreover, anti-foundationalism allows us to reclaim the knowledge of human rights and the agency to develop them further. On the one hand, the normative content of human rights should not, even potentially, be completely alien to our normative practices. If the true normative requirements of human rights are possibly detached from our normative practices, it follows that all those involved in such practices could be completely wrong about the normative requirements of human rights. But what confidence may we then have to presuppose that we are reliable in our judgments about human rights? The foundationalist idea that all those involved in human rights practices can be mistaken about what human rights require is not only metaphysically suspicious, but also normatively dangerous: it would mean that humans may be unaware or incapable of figuring out the precepts of human rights.^59^ On the other hand, if human rights requirements are practice-dependent, that makes them fundamentally non-static: they are constantly reconstructed and reimagined, and thus prone to changes that better reflect contemporary normative commitments. This enables us to see human rights as an ever-developing work in progress of figuring out how best to protect important features of human beings, and to balance this concern with other relevant normative considerations. Once the contingent, non-fixed and historically situated nature of human rights is revealed, this opens a more substantial space for change and adjustment: for example, the requirements of human rights may then expand both in terms of their scope (eg to a more expansive conception of social rights) and in terms of their bearers (eg to non-human animals).^60^

### B. Justification

If the grounds of human rights are ultimately practice-dependent, this has indirect but important consequences for how we may think about their justification. The question of justification concerns the nature and range of normative considerations that ground human rights. Recall that foundationalism limits such considerations to valuable features of human beings to account for the universality of human rights. Foundationalism is thus opposed to the idea of external justificatory pluralism: the view that the normative content of human rights is grounded in multiple normative values, some of which are external to valuable features of human beings.^61^ Because foundationalism holds that valuable features of human beings constitutively determine human rights, it rejects external justificatory pluralism as alien to the very notion of human rights.^62^

However, if human rights are ultimately grounded in social practices, the range of their justificatory grounds cannot be restricted by appeals to the nature of human rights, which is purportedly antecedent to such practice. Instead, the answer to this question needs to be sensitive to the justificatory grounds of human rights embedded in the practice: there is no reason to consider only some justificatory grounds as constitutive if they all play a role in normative judgments about the content of human rights. This is not to say that the issue does not depend on a further normative question of how much weight ought to be ascribed to each justificatory ground in a particular context, or even whether a particular justificatory ground should continue to play a role in the grounding base of human rights; rather, human rights practices will only settle the prior issue of whether certain kinds of normative considerations are necessarily excluded from the grounding base of human rights in virtue of their practice-independent nature.

The practice suggests that a range of normative considerations feeds into the conclusions about the normative content of human rights. Reasons of, for example, democratic self-determination, sovereignty and respect for cultural differences are all already embedded in human rights practice, which ultimately grounds the existence of human rights.^63^ For instance, it is counter-intuitive to think that there are human rights violations that are justified on the basis of democratic self-determination or the principle of sovereignty.^64^ In such cases, it is more accurate to say that there are *no human rights violations in the first place*: these kinds of ‘external’ considerations are typically considered to be important in making normative judgments about human rights requirements.^65^ While the normative significance of the protected feature of human beings is the crucial normative consideration, it is balanced against these other reasons *before* an abstract right is recognised as a human right and *before* a concrete conclusion about the content of a human right is reached in any particular case.^66^

An example of this are rights that are subject to limitations and proportionality analysis. Proportionality analysis appears to suggest that a right may be interfered with but that such interference can be justified by ‘external’ considerations. This seems to indicate that the ‘existence’ of a right is analytically prior to consideration of these external reasons. The conclusion is strengthened by the requirement that limitations must not affect the ‘core’ of a human right.^67^ But a more accurate way of describing this situation is to say that proportionality analysis specifies the content of human rights, and facilitates a normative judgment that a human right ultimately does or does not require something.^68^ Interference simply triggers the analysis that may or may not conclude that a human right is violated. What matters is the ultimate normative conclusion about human rights, and the process of proportionality analysis describes how we arrive at such conclusion: by taking into account the ‘external’ considerations as well.^69^

Even if this is so, and the nature of human rights does not conceptually exclude external forms of justification, there could still be reasons to restrict the justificatory grounds to valuable features of human beings. An important intuition behind the foundationalist conception of justification is that human rights are primarily concerned with protecting human beings and giving expression to their equal value. But this need not entail that the normative content of human rights must be grounded in valuable features of human beings only. In fact, the value of such protected features can only be grasped and appreciated against the background of external justificatory grounds. As Raz explains, because of its exclusive commitment to internal forms of justification, foundationalism cannot account for the threshold beyond which an interference with a valuable feature of human beings does not amount to a human rights violation.^70^ An example is Griffin’s claim that human personhood, understood as normative agency, is the only justificatory ground of human rights.^71^ This ground seems too inclusive: many intrusions on normative agency, such as nudging, do not amount to human rights violations.^72^ The temptation is then to reduce human rights protections to the ‘minimum’ conditions for the exercise of such agency.^73^ But this seems too restrictive: the minimum conditions for agency are satisfied even in circumstances of systemic human rights violations, such as slavery. Griffin extends this ‘minimum’ to include the conditions for a successful realisation of human agency, such as minimal education, information and resources.^74^ However, then there is no principled, agency-based reason to determine the content of human rights in such a limited way.^75^ Successful realisation of human agency could potentially ground all the protections necessary to secure the preconditions for leading a good life, which would go well beyond the special character of human rights guarantees.^76^ And the restriction of human rights protections to this ‘minimum’ also seems normatively ad hoc:^77^ it cannot—*pace* Griffin—‘generate most of the conventional list of human rights’ for it would, for example, exclude much more ambitious and arguably attractive guarantees of social, economic and cultural rights accepted in the practice.^78^ As noted by others, similar problems plague foundationalist accounts in general.^79^

The threshold of human rights protection in fact depends on the consequences of determining the normative content of human rights in a particular manner. The key consequence of protecting human beings in the form of human rights is justification of universal concern for their interests, status or needs.^80^ The value of the features of human beings that are protected by human rights thus needs to override the reasons which count against making such protections a matter of universal concern. There are at least three such competing reasons: state sovereignty, which guarantees the internal autonomy of states even when they are not fully democratic or just; democratic self-determination, which allows states to pursue the democratic preferences of their citizens; and respect for cultural differences, which leaves the space to societies to follow their culturally embedded social norms.^81^ Once these external reasons are introduced, it becomes clearer why certain important moral rights—such as the right that others keep their promises given to us—are not human rights: even systematic violation of such rights is not significant enough to override the reasons of sovereignty, democracy and tolerance, and thus justify international concern. Conversely, paradigmatic human rights—such as the right not to be subject to torture—override these external reasons: international concern and protection of such rights is justified even against the views of sovereign states, democratically elected institutions or different cultural practices. The content of human rights claims is thus not fully fixed by reference to a particular property of human beings, but also by reference to external reasons.^82^

This way of justifying human rights makes them context-specific. There is a range of human rights practices, at different levels of development and maturity, and the requirements of human rights can vary in different contexts. Much depends on the institutional and social position of the actor that is making the judgment about human rights requirements, and the kind of concern or interference that is within their purview.^83^ Because human rights requirements depend on the balance of reasons that obtains from a particular evaluative framework, human rights-based interference may be bolder, and human rights requirements more substantive, in frameworks that share a common evaluative identity and a closer relationship between states.^84^ Conversely, more restricted forms of concern may be apposite where the interference with the valuable features of human beings is less significant and systematic, and occurs in a context where the reasons of respect for cultural differences, democratic-decision making and sovereignty count strongly against it. But this need not undermine the notion that there is a global and overarching practice of human rights.^85^ There is a convergence in practice on both the range of justificatory grounds of human rights and the core protections that qualify as human rights: while the structural features of the notion of human rights are relatively fixed, the concrete requirements of human rights are subject to interpretation and change.^86^

To sum up: anti-foundationalism explains the existence of human rights in virtue of contingent social practices and their justification in virtue of plural normative considerations that are embedded in such practices. There are good reasons to accept the anti-foundationalist account of human rights grounds: it avoids the suspect theoretical claims of foundationalism, retains our ability to know and agency to develop human rights requirements and explains how the threshold of human rights is determined without undermining the normative significance and critical potential of human rights. I now turn to the ECtHR to show how its doctrines may look through the anti-foundationalist lens.

## 3. ECtHR: The Two Doctrines

Two doctrines determine the structural parameters of the ECtHR’s human rights analysis: the margin of appreciation (MoA) and the use of European consensus (EuC). The MoA denotes the regulative and adjudicative space that the Court leaves for domestic authorities to work out the content and requirements of the rights set out in the European Convention on Human Rights (ECHR, the Convention).^87^ The ECtHR is willing to restrict the scope of the MoA granted to contracting states in case there is an emerging EuC on the issue, and to follow the interpretation of a certain right that is preferred by the majority of the states.^88^ These two doctrines enable the so-called evolutive or dynamic interpretation of the Convention that allows the Court to gradually update its understanding of rights, while at the same time supporting the subsidiarity of supranational human rights protection when there is no convergence among the contracting parties on a certain human rights issue.^89^ Human rights foundationalism finds these doctrines deeply problematic.^90^

Because the MoA makes human rights requirements contingent upon divergent social practices in contracting states, foundationalists believe that this doctrine gives up on the universality of human rights. Recall that, for foundationalists, the universality of human rights is based on their practice-independent existence. If the MoA makes human rights requirements practice-dependent, it is then also a threat to the so-understood universality. In this vein, Judge De Meyer famously urged the court to ‘banish’ the concept of MoA from its reasoning because—among other things—it ‘implies’ relativism. In his view, ‘where human rights are concerned, there is no room for a margin of appreciation which would enable the States to decide what is acceptable and what is not’.^91^ Similarly, Benvenisti argues that the MoA is a ‘principled recognition of moral relativism’ and as such ‘at odds with the concept of the universality of human rights’.^92^ And Letsas contends that because the content of human rights is ultimately fixed in a practice-independent way, the MoA is either confused (a substantive decision on human rights requirements cannot be based on the doctrine of MoA, which does not bear any relationship to this fixed content)^93^ or unjustified (substantive human rights requirements are independent from and should outweigh any competing institutional concerns which supposedly justify the MoA).^94^ The crux of this complaint is that the MoA does not fit the foundationalist understanding of human rights: it makes human rights requirements contingent upon local practices, which undercuts the notion that the existence of human rights is practice-independent; moreover—as we shall see—the MoA makes the normative content of rights dependent on considerations external to valuable features of human beings, which undermines the idea that justification of rights is grounded in such valuable features only.

The ECtHR’s reliance on EuC raises similar problems. The consequence of the use of consensus is not deference to local understandings of human rights, but exactly the opposite: bringing these different understandings in line and setting a uniform standard. However, this doctrine too makes human rights requirements contingent upon social practices. Again, Benvenisti is instructive: ‘The adjudicating organ must either adopt a moral standard or defer to a relativistic approach based on a comparative analysis. The [ECtHR] has opted for the latter approach by developing the doctrine of consensus.’^95^ The objection is that the Court makes an adequate normative judgment relative to a contingent framework of evaluation that depends on social practices that exist in the majority of European states. This view is foundationalist: it holds that the justification of human rights must be grounded in practice-independent moral standards, and that there is no conceptual space between such moral standards and self-defeating relativism, which cannot provide such grounding.^96^ In what follows, I will argue that anti-foundationalism better explains these two doctrines, and that it is a deficiency of the foundationalist paradigm that it cannot account for the key doctrines of one of the most prominent human rights regimes in the world.

### A. Margin of Appreciation

The MoA is the primary vehicle through which the ECtHR exercises deference to domestic understandings of human rights. The doctrine was first mentioned in the early days of the Convention when the European Commission of Human Rights applied it in the context of derogations in situations of public emergency.^97^ From there, it found its way into the ECtHR’s reasoning, and it is particularly visible in adjudication on qualified rights, which can be balanced against other rights or a set of legitimate public aims,^98^ and super-qualified rights, which can be balanced against any public aim as long as their core is not affected and where an even broader margin of appreciation is granted.^99^ But the MoA is not reserved for qualified rights only, as the Court has allowed it in relation to duties arising from absolute rights as well.^100^ The MoA also applies to the key analytic steps in human rights adjudication: determination of relevant facts,^101^ defining the scope of the right,^102^ and balancing between the rights and public interests or the rights of others.^103^ Finally, the new Protocol 15 explicitly incorporates the MoA doctrine in the Preamble to the Convention.^104^ The doctrine is clearly central to the adjudicative practice established by the ECHR.

There are several paradigmatic elements of the MoA doctrine. First, it is not neutral about the normative content of human rights: deference to national authorities determines whether the Court will find that a right has been violated. It is sometimes assumed that the least controversial use of the MoA arises in cases where the ECtHR defers on questions of fact.^105^ But expertise- or facts-based deference does not exclude a normative judgment about human rights and their importance in relation to other rights, interests or aims. As the ECtHR put it in *Buckley v UK*, ‘the national authorities … are in principle better placed than an international court to evaluate local needs and conditions’.^106^ The Court thus considers national authorities to be better informed about the local ‘conditions’, which may refer to factual expertise, but deference is also granted on the basis of local ‘needs’, which are a part of the evaluative judgment about the appropriate balance between the protection of human interests and status and the aims of the society in question. For example, in *Hatton and Others v UK*, the ECtHR considered whether the applicants’ rights to family life had been violated by allowing night flights at Heathrow airport. The Court granted the MoA both because the domestic expert scrutiny was conducted properly and because the states are allowed a leeway to make decisions about their economic development.^107^ But the best way to pursue economic development is not a value-free decision of technical or expert nature. It is a value choice which was left to the UK to make, and this implies that the domestic decision did not violate the family life rights under Article 8 ECHR. This shows that the application of the MoA is inseparable from the substantive decision on the normative content of human rights and that, as such, it must follow from a sound conception of human rights grounds.^108^

Secondly, by virtue of its use of the MoA, the Court expresses a position about the existence of human rights. The Court uses the MoA to reconcile the existing diversity of national understandings of rights with the emerging consensus on the European level.^109^ But, in so doing, the Court also makes the content of human rights dependent on contingent social practices. Consider the argument from *Handyside* about the conception of ‘morals’ as one of the possible grounds for limitation of the freedom of expression:^110^

it is not possible to find in the domestic law of the various Contracting States a uniform European conception of morals. The view taken by their respective laws of the requirements of morals varies from time to time and from place to place, especially in our era which is characterised by a rapid and far-reaching evolution of opinions on the subject. By reason of their direct and continuous contact with the vital forces of their countries, State authorities are in principle in a better position than the international judge to give an opinion on the exact content of these requirements …

The idea that underpins the reasoning in *Handyside* is that unless there is a relatively uniform understanding of a particular limitation of a human right in Europe, the states enjoy discretion in determining the standard of its protection. The institutional point—that domestic authorities are better placed to determine whether a limitation is justified—follows from the notion that different local understandings can actually shape the normative requirements of human rights.^111^

Thirdly, the MoA is central to the ECtHR’s understanding of the justification of human rights. The Court’s normative analysis proceeds from the significance of the protected feature of human beings. In this sense, the ECtHR characteristically refers to the ‘importance of the right for the individual’ or to a ‘particularly important facet of an individual’s existence’.^112^ But the MoA doctrine also introduces further considerations, external to the protected feature, as justificatory grounds of human rights. The first is the respect for diverse understandings of human rights in the contracting states. Local evaluative frameworks may be protected even if there is a consensus in Europe. For example, in *A, B and C v Ireland*, the Court found that Ireland’s abortion regime was not incompatible with the Convention despite a significant consensus pulling in the opposite direction; similarly, in *SAS v France*, the Court decided that the ban on face covering in public spaces in France is not in violation of the Convention rights regardless of the fact that a similar ban existed in Belgian law only.^113^ This implies that the centrality of a particular normative commitment to a national evaluative framework is a consideration that determines human rights requirements not only as a consequence of the lack of consensus, but also in its own right. It also intimates that the understanding of the existence of human rights as practice-dependent determines the central parameters of such justification, but that there is a space for the choice and judgment of the Court within the boundaries set by the practice. And such choices and judgments may be inconsistent with other relevant considerations that the practice suggests ought to be taken into account. In other words, the Court may get the balance between different justificatory grounds embedded in the practice established by the ECHR wrong. For example, in both *A, B and C* and *SAS*, the Court arguably failed to protect minorities, and it did not strike a thoughtful balance between their interests and respect for national evaluative frameworks. But the point here is structural: the depth of disagreement and centrality of certain evaluative attitudes to national frameworks partly determine the justification of human rights, regardless of the difficult question about the appropriate balance between competing normative concerns.

The second external justificatory consideration that determines the content of human rights is sovereignty.^114^ The early approach to the balance between the value of sovereignty and protected features of human beings is visible in cases where states derogated from human rights duties in instances of public emergency.^115^ The Commission used the MoA to defer to domestic understanding of justified derogations in situations that ‘threatened the life of a nation’: in other words, where the states struggled to establish their internal sovereignty. While this approach has since been abandoned,^116^ it is theoretically important because it signals that it is difficult to understand the notion and boundaries of human rights without taking into consideration the idea of sovereignty to which human rights impose the most important limits. And the move away from sovereignty-based justifications for human rights derogations is cogent. Because a thicker evaluative community in Europe has been created by virtue of sovereignty-limiting international human rights treaties, a context-sensitive approach to justification of human rights suggests that sovereignty should play a more limited role in determining the content of rights.

Finally, the Court relies on the doctrine of MoA to leave space for democratic decision making. While the ECtHR sees democratic considerations as essential to the justification of rights, it does not succumb to majoritarianism: the Court has repeatedly stated that an inclusive, representative and reflective process in domestic institutions is a reason for granting a wider MoA.^117^ The reasons for deference to the democratic decisions of domestic institutions included, for example, the fact that the domestic decision is a result of ‘an exceptionally detailed examination of the social, ethical and legal implications’ and ‘the fruit of much reflection, consultation and debate’,^118^ that there were opportunities for interested parties to make relevant representations,^119^ and that the political debate showed awareness of the ‘sensitivity’ of a particular issue.^120^ Similarly, when the wide MoA was not granted, the Court for example noted that the domestic legislatures did not seek to ‘weigh the relevant competing individual and public interests or assess the proportionality of the restriction’,^121^ or that they failed to have a ‘substantive debate … of current human rights standards’.^122^ Human rights are thus not understood as pre-political moral requirements that are in an inherent tension with the democratic process; instead, a democratic and inclusive deliberation is the appropriate way to work out the specific content of human rights under the circumstances of pluralism and disagreement.^123^

### B. European Consensus

The doctrine of EuC allows the ECtHR to diverge from local understandings of human rights, develop new standards and change them over time.^124^ According to the ECtHR, the Convention is a ‘living instrument’ and its normative content evolves to fit the needs and conditions of contemporary European societies.^125^ EuC is the primary way of determining whether such change is justified.^126^ This doctrine is used for interpretation of all the rights from the Convention (including absolute rights),^127^ and in relation to both the scope of the right and its potential balancing with other rights and societal interests.^128^ If state action is deemed to be outside the consensus, the ECtHR may conclude that it violates the ECHR.^129^ Again, this doctrine is central to the ECtHR’s practice. But what does it tell us about the ECtHR’s understanding of human rights grounds?

First, EuC grounds the normative content of rights. As the ECtHR explained in *Tyrer*, the Convention ‘must be interpreted in the light of present-day conditions’ and ‘the Court cannot but be influenced by the developments and commonly accepted standards in … the member States’.^130^ The conditions that the Court refers to are not related to new empirical knowledge.^131^ It is the normative requirements of human rights that can change over time. The focus of the Court is on the normative attitudes of ‘acceptance’ of ‘standards’ that are currently prevalent but had not been in the past. This attaches the content of human rights to normative judgments that are produced within contingent and evolving practices. And the EuC doctrine can lead to substantial changes in the content of rights. For example, based on consensus-led evolution of human rights requirements, the Court concluded that a range of practices were no longer acceptable, including corporal punishment for minors,^132^ criminalisation of homosexuality,^133^ discrimination against children based on the marital status of their parents^134^ and denial of official recognition of preferred gender identity.^135^

Secondly, the role of consensus in evolution of human rights requirements suggests that the ECtHR understands them as practice-dependent. If European human rights practices evolve in a particular direction, the content of human rights evolves as well. This is neither to say that the use of consensus does not involve a substantive normative judgment, nor to say that such use is mechanical.^136^ But reliance on EuC conditions the Court’s normative judgments to such an extent that it can only be explained by an anti-foundationalist conception of the existence of human rights. On the one hand, the EuC doctrine is pervasive, and it is the primary vehicle through which the ECtHR changes its interpretation of rights.^137^ On the other hand, it carries an independent normative weight in judicial reasoning, and it is not used as a demonstration that the practice has moved closer to some practice-independent idea of human rights. Let me explain.

If EuC were a mere addition to a substantive judgment about practice-independent content of human rights, it would be redundant and unlikely to play a prominent role in the ECtHR’s judgments. But EuC can lead to a change in direction on a substantive human rights issue in quite a short time. For example, in *Sheffield and Horsham v UK*, because of the lack of consensus, the Court found that not recognising preferred gender on official documents did not violate human rights;^138^ four years later, in *Goodwin*, based on the existence of consensus, it changed its approach completely and found a violation.^139^ It would be difficult to argue that the Court was not aware of substantive normative arguments in *Sheffield*, or that the use of consensus in *Goodwin* was superfluous; rather, the content of human rights changed because a consensus was established. Moreover, the consensus-based reasoning need not lead to more expansive protection of individual interests in relation to societal goals.^140^ Consensus, instead, aims to adjust the content of human rights to currently prevailing evaluative commitments of the contracting states.

The practice-dependent existence of human rights also makes their content context-specific. While one of the main goals set in the Preamble to the Convention was the recognition and enforcement of rights from the Universal Declaration of Human Rights, this was not to be achieved in a way which would not be sensitive to the specific values obtaining on the continent. The Preamble grounds the Convention rights in the ‘common heritage of political traditions’ in Europe. The contracting states committed to participate in the global practice of human rights, but the concrete content of this commitment is not determined through a process of specification of abstract (or application of uniform) values, but by building on existing common values. By establishing the Convention system, the states committed to the protection of values enshrined in the Convention, but they also made a partly content-independent commitment to having a set of common answers to what these values require. When there is a growing consensus or trend on a certain issue, the Court is supporting this commitment to a shared core understanding of human rights requirements, and giving effect to the specific understanding of human rights in European evaluative practices.^141^

Thirdly, this understanding of the existence of human rights does not preclude normative judgments, but determines the parameters within which they are made. Such judgments are conditioned by EuC and the MoA—which disclose the evolving content of human rights in Europe—but are also sensitive to a number of substantive commitments that are implicit in the practice of human rights. It is thus sometimes suggested that the Court not only sees its evolutive and consensus-based judgments as better from the perspective of their fit with the common evaluative practice, but also as all-things-considered better normative judgments. Letsas, for example, argues that ‘it is not enough that a different understanding has evolved, this understanding must also be better, i.e. towards the truth of the substantive protected right’.^142^ Without doubt, the Court does believe that its evolutive interpretations are better than the previous ones. But it is nowhere suggested, nor is it necessary, that they are better in virtue of practice-independent rights or principles.^143^ It is more plausible to think, given the weight given to EuC, that the Court understands its judgments as being better because they make European human rights practice more systematic and coherent. The Court may see the new consensus as an improvement because it brings certain opinions in line with deeper evaluative commitments, thereby achieving a better balance between basic normative intuitions and their generalisations in the form of principles and purposes. The Court may also understand the systematising process that occurs through the use of EuC as contributing to the development of a moral community that is committed to human rights protection, thereby making its overall evaluative outlook stronger and more efficient.^144^ Anti-foundationalism does not preclude the notion that some normative commitments are more important than others: it only points out that they are all (potentially and incrementally) revisable in social practices.^145^ The concrete evaluative commitments that arise in human rights practice need not always be compatible, and the existence of consensus is a signal that a new equilibrium has been reached.^146^

### C. Explanatory and Normative Objections

The interplay between the MoA and EuC reveals an anti-foundationalist conception of human rights. The ECtHR understands the existence of human rights as practice-dependent, their requirements as context-sensitive and their justification as externally pluralistic. But there can be two sets of objections to this analysis. The first suggests that the explanatory force of anti-foundationalism is limited, either because an account of the MoA and EuC need not presuppose anything about the grounds of human rights or because it need not presuppose anti-foundationalism. The second suggests that even if anti-foundationalism is generally plausible and attractive, it would still be normatively better for the ECtHR to conceptualise human rights in foundationalist terms.^147^

The first objection is that the MoA and EuC do not implicate questions about the grounds of human rights. These doctrines could be explained in terms of other normative considerations: for example, under the circumstances of disagreement and uncertainty, the ECtHR could have a reason to defer to the outcomes of more representative processes;^148^ or there could be reasons to sacrifice the protection of human rights in a specific case to avoid a possible backlash that could undermine such protection more generally.^149^ The key to the objection is the distinction between the justificatory grounds of the Court’s deference and the justificatory grounds of human rights.^150^ However, while the objection is based on a plausible account of the reasons for deference, it is not sufficiently sensitive to the nature of legal judgments. The Court makes authoritative decisions about the normative content of human rights, and when such judgments are based on the MoA and EuC, the justifying grounds of these doctrines become a part of the grounds of human rights. The ECtHR’s judgments proclaim whether rights have been violated, not that rights have been violated but the Court lacks legitimacy or capacity to remedy the violation. And the connection between the external justificatory grounds and human rights is not causal, but conceptual: regardless of the motivations that might have contributed to the Court’s decision to defer, the normative content of human rights is still fixed in a particular way by the Court’s judgment based on the MoA and EuC.

A further objection could accept that the MoA and EuC partially ground human rights, but argue that foundationalism can account for these doctrines. For example, the interplay between EuC and the MoA could be explained by the wisdom of crowds argument.^151^ The assumption behind this argument is that the consensus between similarly placed actors will point to a correct decision if they are more likely than not to reach such a decision on their own and to the extent that they are allowed to reason independently from each other.^152^ EuC could represent such a consensus and the MoA could guarantee the necessary degree of independence for an unenforced consensus to emerge.^153^ However, while this argument is insightful, to undermine the anti-foundationalist explanation of the MoA and EuC, it would need to assume that the consensus allows the Court to gain epistemic access into the practice-independent domain of human rights. This is a difficult assumption to sustain. On the one hand, the wisdom of crowds argument is silent on this matter. The argument presupposes that the decision makers must be similar and accept certain common premises: for the model to work in the context of human rights, the decision makers must already share a good deal of evaluative commitments and views about human rights.^154^ The argument thus relies on the overlap in human rights practices, but does not epistemically verify it in relation to practice-independent human rights. On the other hand, the argument better fits the anti-foundationalist paradigm. Because foundationalism presupposes the possibility that practices could be in a radical error in relation to practice-independent human rights, the basic initial agreement necessary for the wisdom of crowds argument to work can also be mistaken. If this is so, the considered consensus that emerges from this initial basic agreement can be equally incorrect. But if human rights are grounded in justificatory considerations embedded in human rights practices—as anti-foundationalism would have it—then the standard of correctness is not attached to practice-independent human rights, and the emerging consensus of decision makers who already accept such justificatory grounds is likely to strike the appropriate balance between them through the process of independent reflection and choice.^155^

The more important critiques suggest that the ECtHR would be normatively mistaken to embrace anti-foundationalism. The key such objection is that the ECtHR’s anti-foundationalism undermines the protection of minorities. But it is not clear that the protection of minorities is weaker as a direct consequence of the ECtHR’s anti-foundationalism. On the one hand, the EuC doctrine does not mean that minorities will be subjected to some pan-European majoritarian understanding of their rights: the use of consensus for evolutive interpretation works in favour of minorities in the vast majority of cases.^156^ And the lack of consensus does not mean that the protection will not be granted. The Court is willing to treat the contracting states’ (contingent and practice-dependent) commitment to minority protection as a reason to relax its criteria for establishing whether there is a consensus on an issue, showing that its understanding of the existence of human rights does not overshadow the array of normative commitments arising in the practice.^157^ On the other hand, the MoA doctrine need not undermine the position of minorities. As already explained, the conception of democratic decision making the Court accepts is not majoritarian and requires an inclusive, representative and reflective process to work out the best way to protect minorities in cases of significant moral disagreement. In such cases, even if there is a European consensus, the Court is willing to grant the states some leeway if the peculiar position they hold is a consequence of their evaluative commitments which have been worked out in the domestic democratic process.

There are many decisions of the Court that could have been more forceful in their protection of minorities. And had the Court thought about human rights in foundationalist terms, it would probably have been more confident in imposing its views on the contracting states. But this would come at a price. That price is the assumption of an epistemically privileged position in relation to a number of actors, including those involved in the democratic process.^158^ The fact that human rights are practice-dependent does not leave them without bite: there is a fair bit of agreement in the practice that some actions clearly violate minority rights. But when it comes to the grey area of disagreement, a case can be made that states should be allowed to exercise not only their own moral judgments, but also their political choice. At the same time, the criticism of the Court is based on the arguments that are available in the practice itself, including the contingent commitment to the protection of minorities.^159^

The other prominent normative objection is that the anti-foundationalist approach erodes the universality of human rights. The ECtHR plays a global role and may set the standard for other human rights institutions and actors. Benvenisti, for instance, thinks that—by virtue of its use of EuC—the Court ‘relinquishes its duty to set universal standards from its unique position as a collective supranational voice of reason and morality’.^160^ The Court indeed needs to be aware of the consequences of its decisions for the rest of the world. But universality of rights does not imply their uniformity. As I explained, universality is best understood as a practical commitment, which suggests that the actor making a decision on human rights requirements would be willing to apply the same standards to all human beings if it were similarly institutionally placed. Such universality is based on the realisation of the central evaluative commitments that an institutional actor is prepared to protect, but these commitments need not be uniform. And if the idea of human rights is about protecting the views of minorities, then such pluralism is conducive and not detrimental to human rights. The possible fragmentation of uniformity is not a sign of undermined universality, but a safeguard against the excessive confidence of any specific conception of human rights. The better view, then, is that the ECtHR should not conceive of itself as a universal voice of reason and morality, but should perform its global role through engagement with other institutions in order to work out what the common and global practice of human rights demands.^161^

It would, of course, be unwise to ignore current authoritarian threats to human rights in this context. But it is not clear that the belief in the ultimate practice-independence of human rights grounds is vital to countering such tendencies. It is possible, in fact, that a more foundationalist outlook of the ECtHR would be seen as a threat to a pluralistic understanding of human rights requirements and lead to a more significant backlash.^162^ And because the foundationalist approach invites the view that human rights matter only if they are ultimately practice-independent, it can inadvertently undermine the commitment to human rights: once the weaknesses of this view are exposed, the whole idea of human rights might be rejected as misguided.^163^ If foundationalism is not presupposed, however, then such a practice-independent status of human rights is not necessary for their protection.^164^ In other words, anti-foundationalism recognises the importance of human rights, but is based on the belief that human practices can provide robust and effective grounds for them. For if we cannot trust ourselves to secure the basic values we believe in, it is unlikely that any philosophical conception will do it for us.

## 4. Conclusion

Let me sum up the argument. I made a distinction between foundationalist and anti-foundationalist approaches to human rights grounds, and argued that anti-foundationalism is theoretically apposite and normatively appealing. I then examined two key doctrines of the ECtHR and concluded that they better fit the anti-foundationalist paradigm. The case was based on the fact that the two central doctrines of the ECtHR—the MoA and EuC—indicate that the Court understands the existence of the ECHR rights as practice-dependent and context-specific, and that it sees their justification as plural and sensitive to considerations external to the protected attributes of human beings. These arguments are mutually reinforcing. On the one hand, there are independent (non-ECtHR-related) reasons to believe that anti-foundationalism is sound and attractive, and the fact that the ECtHR’s practice fits the anti-foundationalist understanding of human rights counts in its favour. On the other hand, the fact that the ECtHR understands human rights grounds in this way is one of the reasons that can be adduced in favour of anti-foundationalism, because it compels the foundationalists to counter-intuitively argue that the participants in one of the most developed human rights practices are mistaken about the nature of human rights.

The central message of this article is that the dominant paradigm of analysis and critique of the MoA and EuC needs to change: these doctrines cannot be rejected on a simple assumption that foundationalism is correct. And if anti-foundationalism is a preferable conception of human rights grounds, then the context-sensitive approach of the Court is generally warranted, both in terms of its respect for the domestic understanding of human rights requirements through the MoA and in terms of its expansion of such requirements in line with EuC.

## Footnotes

1 For foundationalism as a thesis about the ultimate practice-independence of human rights grounds, see eg Richard Rorty, ‘Human Rights, Rationality, and Sentimentality’ in Stephen Shute and Susan Hurley (eds), *On Human Rights* (Basic Books 1993). Foundationalism has also been used to denote the idea that there is one normative value that grounds all human rights: John Tasioulas, ‘Towards a Philosophy of Human Rights’ (2012) 65 CLP 1.

2 Foundationalism is assumed in prominent theoretical accounts of the European Convention on Human Rights (ECHR). See eg Eyal Benvenisti, ‘Margin of Appreciation, Consensus, and Universal Standards’ (1999) 31 International Law and Politics 843; George Letsas, *A Theory of Interpretation of the European Convention on Human Rights* (OUP 2007). A notable exception is Marie-Bénédicte Dembour, *Who Believes in Human Rights? Reflections on the European Convention* (CUP 2006).

3 In other words, anti-foundationalism denies the existence of ‘extra-contextual, ahistorical, and non-situational’ grounds of human rights. Stanley Fish, *Doing What Comes Naturally: Change, Rethoric, and the Practice of Theory in Literary and Legal Studies* (Duke UP 1989) 344.

4 Anti-foundationalism understands human rights requirements as context-specific elaborations of practice-dependent evaluative commitments that can justify certain forms of universal concern based on normatively significant features of human beings, notwithstanding the reasons that may count against such concern. This view shares affinities with the political conceptions of human rights which also ascribe explanatory primacy to human rights practices and accept that the normative grounds of rights are not exhaustively determined by valuable features of human beings. See eg John Rawls, *The Law of Peoples* (Harvard UP 1999); Charles R Beitz, *The Idea of Human Rights* (OUP 2009); Joseph Raz, ‘Human Rights without Foundations’ in John Tasioulas and Samantha Besson (eds), *The Philosophy of International Law* (OUP 2010). But the political conceptions are typically not committed to a further thesis about the ultimate practice-dependent existence of human rights.

5 I will assume that grounding can obtain between both facts and entities. See Jonathan Schaffer, ‘On What Grounds What’ in David Chalmers, David Manley and Ryan Wasserman (eds), *Metametaphysics: New Essays on the Foundations of Ontology* (OUP 2009); Jonathan Schaffer, ‘Grounding, Transitivity, and Contrastivity’ in Fabrice Correia and Benjamin Schnieder (eds), *Metaphysical Grounding: Understanding the Structure of Reality* (CUP 2012) 124.

6 George Kateb, *Human Dignity* (Harvard UP 2011).

7 Alan Gewirth, *Human Rights: Essays on Justification and Applications* (University of Chicago Press 1982); James Griffin, *On Human Rights* (OUP 2008).

8 See eg Fabienne Peter, ‘The Human Right to Political Participation’ (2013) 7 Journal of Ethics & Social Philosophy 2.

9 For different senses of ‘foundations’, see Jeremy Waldron, ‘Is Dignity the Foundation of Human Rights?’ in Rowan Cruft, S Matthew Liao and Massimo Renzo (eds), *Philosophical Foundations of Human Rights* (OUP 2015) 125–37.

10 Jonathan Schaffer, ‘Grounding in the Image of Causation’ (2016) 173 Philosophical Studies 49.

11 Schaffer, ‘On What Grounds What’ (n 5) 351. For example, Griffin argues that ‘Out of the notion of personhood we can generate most of the conventional list of human rights’: Griffin (n 7) 33.

12 Gewirth (n 7) 41; Griffin (n 7); John Tasioulas, ‘On the Foundations of Human Rights’ in Cruft, Liao and Renzo (n 9).

13 A John Simmons, *Justification and Legitimacy: Essays on Rights and Obligations* (CUP 2001) 185.

14 Human rights are seen as ‘intermediate moral principles: mediating between the fundamental values (if any) that ground them … and the institutional and social structure that implements them’: J Tasioulas, ‘On the Nature of Human Rights’ in G Ernst and J Heilinger (eds), *The Philosophy of Human Rights: Contemporary Controversies* (De Gruyter 2012) 57.

15 As Waldron puts it, this view presupposes that each human right is ‘based on some fact about human nature’: Waldron, ‘Is Dignity the Foundation of Human Rights’ (n 9) 120.

16 Simmons (n 13) 185. Griffin also trusts that facts about human rights obtain in a pre-social and pre-institutional state of nature because their justification does not depend on ‘social status or relation’: Griffin (n 7) 50–1.

17 John Tasioulas, ‘Human Rights, Legitimacy, and International Law’ (2013) 58 Am J Juris 1, 2–3.

18 See David Luban, ‘Human Rights Pragmatism and Human Dignity’ in Cruft, Liao and Renzo (n 9) 263; Andrea Sangiovanni, ‘Human Rights Practices’ (2020) Critical Review of International Social and Political Philosophy 1, 8–9 (early view).

19 In this view, the point of the practice of is ‘to *track* human rights, not to *constitute* them’: Sangiovanni, ‘Human Rights Practices’ (n 18) 2. Emphasis in the original. See eg Griffin (n 8) chs 6 and 7; Thomas Nagel, *Concealment and Exposure: And Other Essays* (OUP 2002) 33; John Tasioulas, ‘The Moral Reality of Human Rights’ in Thomas Pogge (ed), *Freedom From Poverty as a Human Right: Who Owes What to the Very Poor?* (OUP 2007) 76–7; Tasioulas, ‘Towards a Philosophy of Human Rights’ (n 1) 26–9.

20 This must hold at least for abstract rights from which other rights are derived. See Griffin (n 7) 50; S Matthew Liao and Adam Etinson, ‘Political and Naturalistic Conceptions of Human Rights: A False Polemic?’ (2012) 9 Journal of Moral Philosophy 327, 336–43.

21 Griffin includes a number of such contingent grounds under the name ‘practicalities’, but in his view they are also ‘not tied to particular times or places’: Griffin (n 7) 38.

22 Tasioulas argues that only the values underpinning moral human rights need to be diachronically universal, as opposed to moral human rights themselves: John Tasioulas, ‘Taking Rights out of Human Rights’ (2010) 120 Ethics 647, 671–2.

23 For example: human agency or autonomy (Gewirth (n 7) and Griffin (n 7)); interests (John Tasioulas, ‘On the Foundations of Human Rights’ (n 12)); or needs (David Miller, ‘Grounding Human Rights’ (2012) 15 Critical Review of International Social and Political Philosophy 207).

24 Normative values that count in favour or against establishing certain protections as human rights, such as the value of democratic self-determination, may ground the normative content of human rights while being ultimately practice-independent and not directly justified by valuable features of human beings. See eg Allen Buchanan, *The Heart of Human Rights* (OUP 2013).

25 See eg the literature cited in n 23.

26 While the distinction between existence and justification is straightforward if grounding relations can be separated into distinct categories (so that, for example, metaphysical, natural and normative grounding are different: see Kit Fine, ‘Guide to Ground’ in Fabrice Correia and Benjamin Schnieder (eds), *Metaphysical Grounding* (Cambridge University Press 2012) 38), the argument that follows is better cashed out in terms of the unity of grounding thesis, which explains grounding as one metaphysical relation (Selim Berker, ‘The Unity of Grounding’ (2018) 127 Mind 729). Foundationalism neglects the complete grounding base for facts about human rights, which include both facts about justification and facts about practices (whereby the latter ground the former).

27 Anti-foundationalism is a philosophical sensibility which is not often clearly articulated, and I define it here in opposition to the two foundationalist theses. While this understanding draws on the views of a number of authors, cited below, it does not claim to be the best reconstruction of any such view in particular.

28 See eg Rorty (n 1).

29 Sangiovanni, ‘Human Rights Practices’ (n 18) 8–9. Furthermore, the practice-dependent normative framework which grounds human rights may well be universal. For example, it could be that the equal value of all human beings is widely and universally accepted.

30 Arendt sees human rights as intersubjective political commitments: ‘We are not born equal; we become equal as members of a group on the strength of our decision to guarantee ourselves mutually equal rights.’ Hannah Arendt, *The Origins of Totalitarianism* (new edn, Hardcourt Brace Jovanovich 1973) 301.

31 This is where anti-foundationalism resembles the political conceptions. See the literature cited in n 4.

32 The ‘appropriateness’ of such balance is also practice-dependent. For the possibility of such practice-based justification, see Luban (n 18) 277–8.

33 A roughly equivalent analysis would apply to the question of their synchronic existence and universality.

34 See eg Jeremy Waldron, ‘Human Rights: A Critique of Raz/Rawls Approach’ in Adam Etinson (ed), *Human Rights: Moral or Political?* (OUP 2018) 117; Joseph Raz, ‘Human Rights in the Emerging World Order’ in Cruft, Liao and Renzo (n 9) 224–5; Sangiovanni, ‘Human Rights Practices’ (n 18) 9. Such claims seem to apply even to rights which appear more central, such as right to life or prohibition of torture, given that the vocabulary and key features of such rights were not recognised at the time.

35 Brian Epstein, *The Ant Trap: Rebuilding the Foundations of the Social Sciences* (OUP 2015) 124.

36 It is possible to say that the right to a fair trial or to free elections of cave-dwellers was not respected. For the limits of such possibilities, see Bernard Williams, *Moral Luck: Philosophical Papers 1973–1980* (CUP 1981) ch 11; Bernard Williams, *Ethics and the Limits of Philosophy* (2nd edn, Routledge 1985) ch 9.

37 Griffin (n 7) 50; Tasioulas, ‘The Moral Reality of Human Rights’ (n 19) 76–7; John Tasioulas, ‘Human Rights, Universality and the Values of Personhood: Retracing Griffin’s Steps’ (2002) 10 European Journal of Philosophy 79; David Miller, ‘Joseph Raz on Human Rights: A Critical Appraisal’ in Cruft, Liao and Renzo (n 9) 238–40.

38 See eg International Covenant on Civil and Political Rights, art 14(1).

39 While some rights may be subject to the condition that only a minimal provision necessary in a particular historical period is guaranteed (see eg Liao and Etinson (n 20) 339, in the context of the right to education), for other rights even such a minimum would need to include protections which are similar to contemporary rights (eg a right to political participation could have been similar in previous eras).

40 Raz, ‘Human Rights in the Emerging World Order’ (n 34) 225. See also Andreas Føllesdal, ‘Theories of Human Rights: Political or Orthodox—Why It Matters’ in Reidar Maliks and Johan Karlsson Schaffer (eds), *Moral and Political Conceptions of Human Rights* (CUP 2017) 85.

41 There is a significant discontinuity between the understanding of the normative content and structure of rights in different historical eras, and it is questionable if we can meaningfully consider them to be mere interpretations of the same abstract idea. See eg Samuel Moyn, *The Last Utopia: Human Rights in History* (Harvard UP 2010); Samuel Moyn, ‘Human Rights in Heaven’ in Etinson (n 34) 74–8. For the historical origins of the contemporary idea of human rights, see Steven Wheatley, *The Idea of International Human Rights Law* (OUP 2019); Jack Donnelly, *Universal Human Rights in Theory and Practice* (2nd edn, Cornell UP 2003) ch 5.

42 While this may seem similar to other domains, such as mathematics, where we might have previously failed to access some practice-independent facts through the process of reasoning (Thomas Scanlon,‘The Appeal and Limits of Constructivism’ in James Lenman and Yonathan Shemmer (eds), *Constructivism in Practical Philosophy* (OUP 2012) 226–7), these domains are in fact not analogous. First, the mistakes in the domain of mathematics never pertained to the most central features or axioms which are disputed in the domain of human rights (eg whether 2+2=4 has not been disputed, while whether dignity or freedom ground human rights still is). Secondly, mathematical facts play a crucial role in scientific explanations while facts about human rights do not (see generally Gilbert Harman, *The Nature of Morality: An Introduction to Ethics* (Oxford University Press 1977) ch 1). As Gibbard puts it, ‘at least for arithmetic and geometry, mathematics is part and parcel of empirical knowledge’, and the fact that we have knowledge of mathematics is one ‘aspect’ of our empirical abilities: Allan Gibbard, *Reconciling Our Aims: In Search of Bases for Ethics* (OUP 2008) 21. Even when mathematical facts do not figure in scientific explanations, they presuppose the truth of and are continuous with mathematical facts that do figure in scientific explanations, and they may come to be scientifically useful in future. One could, of course, question whether scientific explanations are ontologically determinative, *pace* WV Quine, ‘Two Dogmas of Empiricism’ (1951) 60 The Philosophical Review 20, but it seems that this, at the very least, puts the burden of proof on those who claim that the domains of human rights and mathematics or science are analogous (more generally on how abstract entities in mathematics exist, see Hilary Putnam, *Philosophy of Logic* (Harper & Row 1971) chs 7 and 8). Finally, while there is disagreement in both sciences and mathematics, there is a much more robust agreement on the process by virtue of which the (presumably) practice-independent facts are verified. See Jeremy Waldron, *Law and Disagreement* (OUP 1999) ch 8.

43 As Williams puts it, ‘unlike the situation with the sciences … there is in the moral case no story about the subject matter and about these past people’s situation which explains why those people got it wrong about that subject matter’. Bernard Williams, ‘Human Rights and Relativism’, in *In the Beginning Was the Deed: Realism and Moralism in Political Argument* (Princeton UP 2005) 66–7.

44 Beitz (n 4) 56.

45 Waldron, *Law and Disagreement* (n 42) ch 8.

46 John Finnis, *Natural Law and Natural Rights* (OUP 1980) ch 3.

47 I discuss this issue at length in Bosko Tripkovic, *The Metaethics of Constitutional Adjudication* (OUP 2017) ch 5.

48 In the context of morality, see eg David Copp, ‘Darwinian Skepticism about Moral Realism’ (2008) 18 Philosophical Issues 186; for a rebuttal, see Sharon Street, ‘Reply to Copp: Naturalism, Normativity, and the Varieties of Realism Worth Worrying About’ (2008) 18 Philosophical Issues 207.

49 If the questions about verification of values were treated as normative questions themselves, this could lead to a conclusion that normative values are the most fundamental grounds of human rights. See Ronald Dworkin, ‘Objectivity and Truth: You’d Better Believe It’ (1996) 25 Philosophy and Public Affairs 87. For the opposite view, based on a more sophisticated account of metaphysical grounding, see Berker (n 26) 771–3.

50 See Williams, ‘Human Rights and Relativism’ (n 43) 65–8.

51 David Lyons, ‘Ethical Relativism and the Problem of Incoherence’ (1976) 86 Ethics 107.

52 David Wong, *Natural Moralities: A Defense of Pluralistic Relativism* (OUP 2006).

53 For the opposite view, see Adam Etinson, ‘On Being Faithful to the “Practice”: A Response to Nickel’ in Etinson (n 34).

54 Beitz (n 4) 105.

55 ibid 127.

56 Gilbert Harman, ‘Moral Relativism’ in Gilbert Harman and Judith Jarvis Thomson (eds), *Moral Relativism and Moral Objectivity* (Blackwell 1996) 43.

57 Williams, *Ethics and the Limits of Philosophy* (n 36) 159.

58 As Ignatieff puts it: ‘An antifoundational humanism may seem insecure, but it does have the advantage that it cannot justify inhumanity on foundational grounds.’ Michael Ignatieff, ‘Human Rights as Idolatry’ in Amy Gutmann (ed), *Human Rights as Politics and Idolatry* (Princeton UP 2001) 88.

59 Sharon Street, ‘Coming to Terms with Contingency: Humean Constructivism about Practical Reason’ in Lenman and Shemmer (n 42) 55–7.

60 For more on this, see Tripkovic, *The Metaethics of Constitutional Adjudication* (n 47) 173–90.

61 Buchanan (n 24) ch 2. It is open to foundationalism to argue that a plurality of valuable features of human beings bear on justification of human rights. Tasioulas, ‘Towards a Philosophy of Human Rights’ (n 1) 26.

62 Tasioulas talks about this question as being fixed by the ‘constitutive nature’ of rights (Tasioulas, ‘Towards a Philosophy of Human Rights’ (n 1) 18), and Gewirth speaks of this view as ‘true by definition’ (Gewirth (n 7) 41).

63 For an elaboration, see Beitz (n 4) chs 5–6.

64 Raz, ‘Human Rights without Foundations’ (n 4) 327–37.

65 See eg Beitz (n 4) chs 5–6; Ignatieff (n 58).

66 In the context of the Universal Declaration of Human Rights, see eg Joe Hoover, ‘Rereading the Universal Declaration of Human Rights: Plurality and Contestation, Not Consensus’ (2013) 12 Journal of Human Rights 217.

67 For instance, the ECtHR refers to the ‘very essence’ of a right that cannot be abridged. See eg *Winterwerp v Netherlands* (1979–80) 2 EHRR 387 para 60; *Young, James and Webster v UK* (1981) 4 EHRR 38 paras 52, 56–7; *Sibson v UK* (1994) 17 EHRR 193 para 29; *Ashingdane v UK* (1985) 7 EHRR 528 paras 57 and 59; *Philis v Greece* (1991) 13 EHRR 741 paras 59 and 65; *Baka v Hungary* (2017) 64 EHRR 6 para 121.

68 For the view that fixing the ‘core’ or the ‘very essence’ involves proportionality analysis, see Robert Alexy, *A Theory of Constitutional Rights* (OUP 2002) 193; Julian Rivers, ‘Proportionality and Variable Intensity of Review’ (2006) 65 CLJ 174, 186–7; Aharon Barak, *Proportionality: Constitutional Rights and Their Limitations* (CUP 2006) 498.

69 On how such concrete intuitions feed into general ideas about human rights, see Rorty (n 1) 116–17; in the context of reflective equilibrium, see Føllesdal, ‘Theories of Human Rights’ (n 40) 87–8.

70 Raz, ‘Human Rights without Foundations’ (n 4) 324–7.

71 Griffin (n 7) 32–3.

72 For other examples and discussion, see Raz, ‘Human Rights without Foundations’ (n 4) 325.

73 Griffin (n 7) 34.

74 ibid.

75 Raz, ‘Human Rights without Foundations’ (n 4) 325–7.

76 ibid 327. Foundationalists claim that their accounts are faithful to human rights practice, which does understand human rights as exceptional in this sense: see eg John Tasioulas, ‘Are Human Rights Essentially Triggers for Intervention?’ (2009) 4 Philosophy Compass 938, 939; Griffin (n 7) 29.

77 The only reason is that human rights protections must somehow be minimal in order to be meaningful, but—if there is a good justification for extensive protection—this assumption does not hold. See Griffin (n 7) 34.

78 ibid 33. For example, the requirements from the International Covenant on Economic, Social and Cultural Rights are far from ‘minimal’: they demand from the states that they commit ‘the maximum of its available resources’ to realisation of these rights (art 2(1)), ‘the widest possible protection and assistance’ to the family (art 10(1)) or ‘the highest attainable standard of physical and mental health’ (art 12 (1)), etc.

79 Andrea Sangiovanni, ‘Beyond the Political–Orthodox Divide: The Broad View’ in Etinson (n 34) 179–82.

80 ibid 185–98.

81 See the literature cited in n 4.

82 Sangiovanni, ‘Beyond the Political–Orthodox Divide: The Broad View’ (n 79) 186.

83 Føllesdal, ‘Theories of Human Rights’ (n 40) 81–6.

84 Raz, ‘Human Rights without Foundations’ (n 4) 331. This does not mean that there is no common and global human rights project. See Andrea Sangiovanni, ‘Beyond the Political–Orthodox Divide: The Broad View’ (n 79) 196–8. For the opposite view, see Victor Tadros, ‘Rights and Security for Human Rights Sceptics’ in Cruft, Liao and Renzo (n 9) 443–7.

85 Sangiovanni, ‘Beyond the Political–Orthodox Divide: The Broad View’ (n 79) 197.

86 Beitz (n 4) 106–17.

87 Howard Charles Yourow, *The Margin of Appreciation Doctrine in the Dynamics of European Human Rights Jurisprudence* (Brill/Nijhoff 1996); Andrew Legg, *The Margin of Appreciation in International Human Rights Law: Deference and Proportionality* (OUP 2012).

88 Kanstantsin Dzehtsiarou, *European Consensus and the Legitimacy of the European Court of Human Rights* (CUP 2015) chs 2–4.

89 Eirik Bjorge, ‘The Convention as a Living Instrument: Rooted in the Past, Looking to the Future’ (2017) 36 HRLJ 243.

90 Letsas argues that the purpose of the ECtHR is ‘to discover … the moral truth about … fundamental rights’ (George Letsas, ‘Strasbourg's Interpretive Ethic: Lessons for the International Lawyer’ (2010) 21 EJIL 509, 540) or ‘what these human rights always meant to protect’ (George Letsas, ‘The ECHR as a Living Instrument: Its Meaning and Legitimacy’ in Andreas Føllesdal, Birgit Peters and Geir Ulfstein (eds), *Constituting Europe: The European Court of Human Rights in a National, European and Global Context* (CUP 2013) 125). This is to be done on the basis of ‘fact-independent moral values’ (‘Strasbourg’s Interpretive Ethic’ 512), which do not ‘depend on institutional recognition or communal acceptance’ (ibid 539). For a critique, see Alain Zysset, ‘Searching for the Legitimacy of the European Court of Human Rights: The Neglected Role of “Democratic Society”’ (2016) 5 GlobCon 16, 22 and 44–5.

91
*Z v Finland* (1997) 25 EHRR 371, dissenting opinion of Judge De Meyer.

92 Benvenisti (n 2) 844.

93 George Letsas, ‘Two Concepts of the Margin of Appreciation’ (2006) 26 OJLS 705, 709–15.

94 ibid 720–9.

95 Benvenisti (n 2) 851.

96 Explaining the EuC away as an unnecessary addition to a substantive moral judgment would disregard the persistent practice of the Court. See eg Letsas, ‘Strasbourg’s Interpretive Ethic’ (n 90) 527–32.

97 ECHR, art 15. See eg *Greece v UK* (1958) DR 181, para 318; *Denmark v Greece* (1969) 12 YB ECHR 1, para 114; *Lawless v Ireland* (1960–61) Series B no 56, para 90.

98 ECHR, arts 8–11.

99
*Yumak and Sadak v Turkey* (2009) 48 EHRR 4.

100
*MC v Bulgaria* (2005) 40 EHRR 20, para 154. This chiefly relates to positive duties, but deference in relation to negative duties occurs at the level of defining the right (eg the ‘minimum level of severity’ in the context of art 3) and not via limitations. See more in Legg (n 87) 204–11.

101 Determination of facts often includes making a normative judgment. The Court often holistically determines whether the decisions of domestic courts are ‘neither arbitrary nor manifestly unreasonable’. See eg *Ndidi v UK* [2017] ECHR 781 para 76.

102 See eg *Oliari and Others v Italy* (2017) 65 EHRR 26; *A, B and C v Ireland* (2011) 53 EHRR 13; *Vo v France* (2005) 40 EHRR 12.

103 See generally Pieter van Dijk and others, *Theory and Practice of the European Convention on Human Rights* (5th edn, Intersentia 2018).

104 Protocol No 15 amending the Convention on the Protection of Human Rights and Fundamental Freedoms, 24 June 2013, art 1, ETS 213 (entered into force on 1 August 2021).

105 Yuval Shany, ‘Toward a General Margin of Appreciation Doctrine in International Law’ (2006) 16 EJIL 907, 918–19. See also *Aksoy v Turkey* (1997) 23 EHRR 553, para 68.

106
*Buckley v UK* (1996) 23 EHRR 101, para 74.

107
*Hatton and Others v UK* (2002) 34 EHRR 1, paras 125–9.

108 Letsas, ‘Two Concepts of the Margin of Appreciation’ (n 93).

109 Føllesdal, ‘Appreciating the Margin of Appreciation’ in Etinson (n 34).

110 See also *Belgian Linguistic Case* (1968) 1 EHRR 252, para 10.

111
*Dickson v UK* (2008) 46 EHRR 41, para 78.

112 See eg *S and Marper v UK* (2009) 48 EHRR 50, para 102.

113
*A, B and C* (n 102); *SAS v France* (2015) 60 EHRR 11.

114 Raz, ‘On Waldron’s Critique of Raz on Human Rights’ in Etinson (n 34).

115 See n 97.

116
*Golder v UK* (1975) 1 EHRR 524, para 40.

117 See eg *Odièvre v France* (2004) 38 EHRR 43, para 49; *Animal Defenders International v UK* (2013) 57 EHRR 21, para 108.

118
*Evans v UK* (2008) 46 EHRR 34, para 86.

119
*Hatton and Others* (n 107) paras 128–9.

120
*Murphy v Ireland* (2004) 38 EHRR 13, para 73.

121
*Dickson* (n 111) para 83. See also *Alajos Kiss v Hungary* (2013) 56 EHRR 38, para 41.

122
*Hirst v UK (No 2)* (2006) 42 EHRR 41, para 79.

123 Richard Bellamy, ‘The Democratic Legitimacy of International Human Rights Conventions: Political Constitutionalism and the European Convention on Human Rights’ (2015) 25 EJIL 1019.

124 Kanstantsin Dzehtsiarou, ‘European Consensus and the Evolutive Interpretation of the European Convention on Human Rights Legitimacy and the Future of the European Court of Human Rights’ (2011) 12 German Law Journal 1730.

125
*Hirsi Jamaa and Others v Italy* (2012) 55 EHRR 21, para 175.

126 The word ‘trend’ captures this notion better than ‘consensus’. See eg *X, Y and Z v UK* (1997) 24 EHRR 143, para 40; *MC* (n 100) para 156.

127
*Pretty v UK* (2002) 35 EHRR 1, para 48; *Opuz v Turkey* (2010) 50 EHRR 28, para 138; *Selmouni v France* (1999) 29 EHRR 403, paras 96–100; *MSS v Belgium and Greece* (2011) 53 EHRR 2, para 251.

128 Christian Djeffal, ‘Consensus, Stasis, Evolution: Consensus, Stasis, Evolution: Reconstructing Argumentative Patterns in Evolutive ECHR Jurisprudence’ in Panos Kapotas and Vassilis P Tzevelekos (eds), *Building Consensus on European Consensus: Judicial Interpretation of Human Rights in Europe and Beyond* (CUP 2019).

129 The Court can find a violation even if there is no consensus. See eg *Hirst* (n 122) para 82.

130
*Tyrer v UK* (1978) 2 EHRR 1, para 31.

131 The Court sometimes defers to the ‘consensus of experts’. See eg *L and V v Austria* (2003) 36 EHRR 55, para 47.

132
*Tyrer* (n 130).

133
*Dudgeon v UK* (1981) 4 EHRR 149, para 60.

134
*Marckx v Belgium* (1979) 2 EHRR 330, para 41.

135
*Goodwin v UK* (2002) 35 EHRR 18, para 84.

136 Fiona de Londras and Kanstantsin Dzehtsiarou, ‘Managing Judicial Innovation in the European Court of Human Rights’ (2015) 15 HRLR 523.

137 Dzehtsiarou, *European Consensus and the Legitimacy of the European Court of Human Rights* (n 88) 21–3.

138
*Sheffield and Horsham v UK* (1999) 27 EHRR 163, para 55.

139
*Goodwin* (n 135) para 90. See also *Bayatyan v Armenia* (2012) 54 EHRR 15, para 102.

140 See eg *Scoppola v Italy (No 3)* (2013) 56 EHRR 19, reverting *Frodl v Austria* (2011) 52 EHRR 5.

141 The Court’s reliance on global developments to strengthen its consensus-based analysis does not make its understanding of human rights practice-independent (*pace* George Letsas, ‘The Truth in Autonomous Concepts: How To Interpret the ECHR’ (2004) 15 EJIL 279). For example, in *Marckx* (n 134), the Court referred to international trends once it established that there was a growing European consensus ‘in the domestic law of the great majority of the member States’, and it did not tie human rights to some practice-independent normative requirements, but to the ‘evolution of rules and attitudes’ (para 141). The Court simply sees European human rights practice as a part of the global human rights practice (see eg *Mamatkulov and Askarov v Turkey* (2005) 41 EHRR 25; Dzehtsiarou, *European Consensus and the Legitimacy of the European Court of Human Rights* (n 88) 45–9).

142 Letsas, ‘The Truth in Autonomous Concepts’ (n 141) 302.

143 The use of ‘autonomous concepts’ doctrine does not commit the Court to foundationalism (*pace* Letsas, ‘The Truth in Autonomous Concepts’ (n 141) 281–5). The doctrine means that the Court is not accepting technical terms from domestic law as determinative of its concepts (see more in Eirik Bjorge, *Domestic Application of the ECHR: Courts as Faithful Trustees* (OUP 2015) ch 7), but this does not entail that such concepts have practice-independent meaning; the Court identifies the paradigmatic elements of a concept based on its use in European states, and by looking at the (practice-dependent) purpose of a particular human rights provision. See eg *Engel and Others v Netherlands* (1976) 1 EHRR 647, para 82). Moreover, this doctrine is limited and pertains mainly to technical terms: Sionaidh Douglas-Scott, ‘Borges’ *Pierre Menard, Author of the Quixote* and the Idea of a European Consensus’ in Panos Kapotas and Vassilis P Tzevelekos (eds), *Building Consensus on European Consensus: Judicial Interpretation of Human Rights in Europe and Beyond* (CUP 2019).

144 Rorty (n 1) 116–17.

145 Anti-foundationalism is not a claim about there being ‘no foundations, but that whatever foundations there are … have been established … in the course of argument and counter-argument on the basis of examples and evidence that are themselves cultural and contextual. Anti-foundationalism … is a thesis about how foundations emerge’: Fish (n 3) 29–30.

146 Føllesdal, ‘Theories of Human Rights’ (n 40) 87–8.

147 For an analogous argument in ethics, see Simon Blackburn, *Ruling Passions: A Theory of Practical Reasoning* (OUP 1998).

148 For a range of arguments along these and similar lines, see Dzehtsiarou, *European Consensus and the Legitimacy of the European Court of Human Rights* (n 88) ch 6; Shany (n 105) 919–22; Janneke Gerards, ‘Pluralism, Deference and the Margin of Appreciation Doctrine’ (2011) 17 ELJ 80, 102–14; Jean-Paul Costa, ‘On the Legitimacy of the European Court of Human Rights’ Judgments’ (2011) 7 EuConst 173; Dominic McGoldrick, ‘A Defence of the Margin of Appreciation and an Argument for its Application by the Human Rights Committee’ (2016) 65 ICLQ 21; Matthew Saul, ‘The European Court of Human Rights’ Margin of Appreciation and the Processes of National Parliaments’ (2015) 15 Human Rights Law Review 745.

149 Shai Dothan, ‘Judicial Deference Allows European Consensus to Emerge’ (2018) 18 Chicago Journal of International Law 393, 407–10; de Londras and Dzehtsiarou (n 136) 534–41.

150 Legg (n 87) 58 and 195.

151 See Dothan (n 149).

152 For an analogous argument in relation to the consensus in foreign law, see Cass R Sunstein, *A Constitution of Many Minds: Why the Founding Document Doesn’t Mean What It Meant Before* (Princeton UP 2009) ch 8.

153 Dothan (n 149) 400–13.

154 ibid 401.

155 I deal with this issue comprehensively in Bosko Tripkovic, ‘The Morality of Foreign Law’ (2019) 17 ICON 732, 741–4 and 750–1. Another objection could be that the MoA-based deference to democratic institutions can be explained by their democratic credentials even if rights are understood in the foundationalist sense. However, democratic considerations only partly explain the MoA, as the ECtHR uses it to defer to a range of other institutions as well (such as domestic courts). Moreover, the anti-foundationalist explanation is more comprehensive and thus preferable: it accounts for both the MoA and EuC.

156 See eg *Bayatyan* (n 139) (religious minorities); *Vinter and Others v UK* (2016) 63 EHRR 1 (criminal offenders); *Dudgeon* (n 133) (homosexuals).

157 See eg *Goodwin* (n 135).

158 Bellamy (n 123) 1022.

159 Donnelly (n 41) 20–1.

160 Benvenisti (n 2) 852.

161 See eg Charles Taylor, ‘Conditions of an Unforced Consensus on Human Rights’ in Joanne R Bauer and Daniel Bell (eds), *The East Asian Challenge for Human Rights* (CUP 1999).

162 See eg David Kennedy, *The Dark Sides of Virtue: Reassessing International Humanitarianism* (Princeton UP 2004) 327–58.

163 For an explanation of how this may transpire, see John L Mackie, *Ethics: Inventing Right and Wrong* (2nd edn, Penguin 1990) 34.

164 Philippa Foot, ‘Morality as a System of Hypothetical Imperatives’ (1972) 81 The Philosophical Review 305, 315–16.

